# Global transcriptome analysis of *Bacillus cereus *ATCC 14579 in response to silver nitrate stress

**DOI:** 10.1186/1477-3155-9-49

**Published:** 2011-11-10

**Authors:** Malli Mohan Ganesh Babu, Jayavel Sridhar, Paramasamy Gunasekaran

**Affiliations:** 1 Department of Genetics, Centre for Excellence in Genomic Sciences, School of Biological Sciences, Madurai Kamaraj University, Madurai - 625 021, Tamil Nadu, India; 2 UGC-Networking Resource Centre in Biological Sciences, School of Biological Sciences, Madurai Kamaraj University, Madurai - 625 021, Tamil Nadu, India

**Keywords:** silver nitrate stress, silver nanoparticles, transcriptomics, *Bacillus cereus*, *sRNA*

## Abstract

Silver nanoparticles (AgNPs) were synthesized using *Bacillus cereus *strains. Earlier, we had synthesized monodispersive crystalline silver nanoparticles using *B. cereus *PGN1 and ATCC14579 strains. These strains have showed high level of resistance to silver nitrate (1 mM) but their global transcriptomic response has not been studied earlier. In this study, we investigated the cellular and metabolic response of *B. cereus *ATCC14579 treated with 1 mM silver nitrate for 30 & 60 min. Global expression profiling using genomic DNA microarray indicated that 10% (n = 524) of the total genes (n = 5234) represented on the microarray were up-regulated in the cells treated with silver nitrate. The majority of genes encoding for chaperones (GroEL), nutrient transporters, DNA replication, membrane proteins, etc. were up-regulated. A substantial number of the genes encoding chemotaxis and flagellar proteins were observed to be down-regulated. Motility assay of the silver nitrate treated cells revealed reduction in their chemotactic activity compared to the control cells. In addition, 14 distinct transcripts overexpressed from the 'empty' intergenic regions were also identified and proposed as stress-responsive non-coding small RNAs.

## Background

Metal nanoparticles exhibit unique electronic, magnetic, catalytic and optical properties that are different from those of bulk metals. Nanoparticles are synthesized using several physical and chemical methods such as laser irradiation, micelle, sol-gel method, hydrothermal and pyrolysis. Attempts are being made to develop nontoxic and environmental friendly methods for the production of metal nanoparticles using biological systems. The use of bacteria, fungi and yeast for the synthesis of metallic nanoparticles is rapidly gaining importance due to the success of microbial production of nanometals [1]. Heavy metals are essential as trace elements and they are found in high concentrations in marine environments, industrial effluents including mining and electroplating industries. Untreated effluents from these industries have an adverse impact on the environment.

Metal ions play important roles in microbial metabolism. Some metal ions are essential as cofactor in the metabolic reactions, others are oxidized or reduced to derive metabolic energy, while heavy metal ions such as Ag^+^, Cd^2+^, Hg^2+^, Co^2+^, Cu^2+^, Ni^2+^, Zn^2+ ^cause toxic effects. To counter the toxic effects, microorganisms have evolved adaptive mechanisms to survive under metal ionic stress [2]. Bioremediation approach is getting more attention because of its economical and environmental friendly aspects. Metal contaminated industrial sites are bioremediated by stimulating indigenous microbial communities. Bacteria belonging to different genera such as *Bacillus*, *Pseudomonas, Escherichia *and *Desulfovibrio *have been shown to accumulate and reduce various heavy metals [3-5]. Ionic silver (Ag^+^) is known to be effective against wide range of microorganisms and has been traditionally used in therapeutics [6]. Basically, silver ions are charged atoms (Ag^+^), whereas silver nanoparticles are zerovalent crystals of nanosize (nm). The crystallized nanoparticles have been used as a source of Ag^+ ^ions in many commercial products, such as food packaging, odour resistant textiles, household appliances and medical devices. Despite growing concerns, little is known about the potential impacts of silver nanoparticles on human health and environment. Microbial resistance to silver is most likely to occur in environments where silver is routinely used; for example, burns units in hospitals, catheters (silver-coated) and dental setting (amalgams contain 35% silver). In spite of the fact that silver is known to exhibit bactericidal effect, its impact on the transcriptome and cellular physiology have not been studied [7-9].

Microorganisms have evolved adaptive mechanisms to face the challenges under silver ionic stress condition. *B. cereus *efficiently precipitates silver as discrete colloidal aggregates at the cell surface and occasionally in the cytoplasm, thus the organism has the ability to reduce 89% of the total Ag^+ ^and remove from the solution [10]. Similarly, *B. licheniformis *[11,12], *B. cereus *PGN1 [13], *B. subtilis *[14] were shown to accumulate silver nanoparticles with well defined size and shape, within the cytoplasm. Inside the cell, the toxic effects of heavy metals include nonspecific intracellular complexation with particularly vulnerable thiol groups. Previous studies reported that several heavy metals were toxic to cellular processes. In Gram-negative bacteria, heavy metal ions can bind to glutathione and the resulting products tend to react with molecular oxygen to form oxidized bis-glutathione, releasing the metal cation and hydrogen peroxide. Some metal ions structurally mimic physiologically important molecules. Some metals are reduced intracellularly by both enzymatic and non-enzymatic reactions. This process may inadvertently cause damage to many cellular components, including DNA and proteins. In addition, metal stress is associated with oxidase activity, biofilm formation, motility, oxidative stress or sulphur assimilation in various microorganisms [12,15]. However, the response exhibited by *B. cereus *at transcript level under silver ionic stress has not yet been studied.

The transcriptional response of *Bacillus *spp. to environmental perturbations can be large and complex, involving multiple transcription factors and their regulons. DNA microarrays of *Bacillus *spp. were already employed to study the global response under acid/base [16], peroxide [17], salt [18,19], organic/inorganic acid shocks [20], metal ions [21], superoxide radicals [22] and bile salts [23] stress conditions. Previously, some effector proteins in *B. subtilis *against multiple metal ion stresses were identified using DNA microarrays, but they were not studied for the global response against the metal ion stress. The availability of complete genome sequence of *B. cereus *ATCC14579 [NC_004722] [24] facilitates to design genome arrays which could be used for the analysis of global transcriptome in response to different stress conditions.

Recent studies have identified non-coding small RNAs (sRNAS) to play vital role in response to a variety of stress conditions. But very few small RNAs were reported in *B. cereus *ATCC14579 [25]. To search for additional sRNAs expressing in response to silver metal stress, we have included those 900 'empty' intergenic regions in the genomic microarray to detect transcripts arising from 'empty' intergenic regions of *B. cereus*. In this study, we performed DNA microarray for genome-wide transcriptional analysis of *B. cereus *ATCC 14579 in response to silver nitrate.

## Results and Discussion

### Effect of silver nitrate on the viability of *B. cereus*

**Figure 1:**
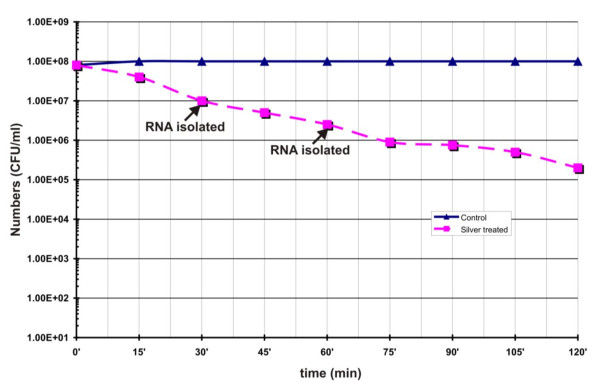
**Survival of *B. cereus *cells during silver nitrate induced stress condition**. Logarithmically grown cells were treated with and without 1 mM AgNO_3_. At intervals samples were withdrawn, suitably diluted and plated on LB agar plate without silver nitrate and incubated overnight at 37°C. The colony forming units (CFU) were determined and plotted against time.

The effect of silver nitrate induced stress on the growth of *B. cereus *ATCC14579 was studied by challenging the culture with silver nitrate. Figure 1 shows the viability of *B. cereus *upon treatment with 1 mM silver nitrate. Exposure to silver nitrate decreased viability of the cells. Within 120 min exposure to silver nitrate, the viable cell number was decreased by two log scale i.e. from 10^8 ^to 10^6 ^cfu/ml. These results suggested that silver nitrate treatment significantly affected the cell viability and growth of *B. cereus *ATCC14579.

### Characteristics of silver nanoparticles formed in *B. cereus*

**Figure 2:**
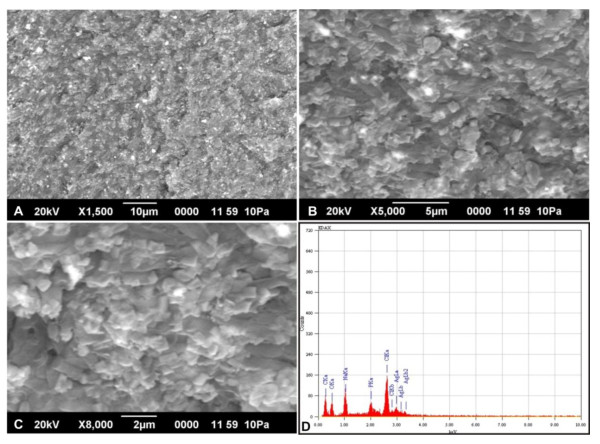
**SEM with EDX analysis of silver nanoparticles synthesized by *B. cereus *ATCC 14579**. Scanning Electron microscopy image (A - C) represents silver nanoparticles synthesised from *B. cereus *ATCC 14579 incubated at 37°C for 1 h. Figure 2D represents the Energy-Dispersive X-ray microanalysis of silver nanoparticles.

Scanning electron microscopy (SEM) analysis of the cells treated for 60 min with silver nitrate revealed the presence of silver nanoparticles within the cells (Figure 2A-C). Energy dispersive X-ray microanalysis (EDX) was done for qualitative analysis of the thin sections from the selected preparations. Elemental analysis of the silver nanoparticles was performed using EDX in SEM. The EDX spectrum of the silver nanoparticles synthesized by *B. cereus *is shown in Figure 2D. The vertical axis displays the number of x-ray counts whilst the horizontal axis displays energy (keV). The peaks between 3.00 - 3.40 keV correspond to the binding energy of Ag_La_, Ag_Lb _and Ag_Lb2 _with ~ 50 - 60 counts, while the peaks near binding energies of 0.3 keV and 0.52 keV belongs to carbon and oxygen respectively. The carbon and oxygen peaks in the EDX analyses can be attributed to the surrounding residual material and/or the carbon tape used for SEM grid preparation. Throughout, the scanning range of binding energies, some peaks belonging to Na, Cl and P were also detected [26,27]. SEM with EDX analysis of the colloids in the cell pellet indicated the presence of silver material in nano size diameters bound within the cell wall of the bacteria. These particles are in the monodispersed size range between 4- 5 nm and spherical shape, which is comparable with silver nanoparticles synthesised by other bacteria [13].

### Microarray experiments and their efficacy

**Figure 3:**
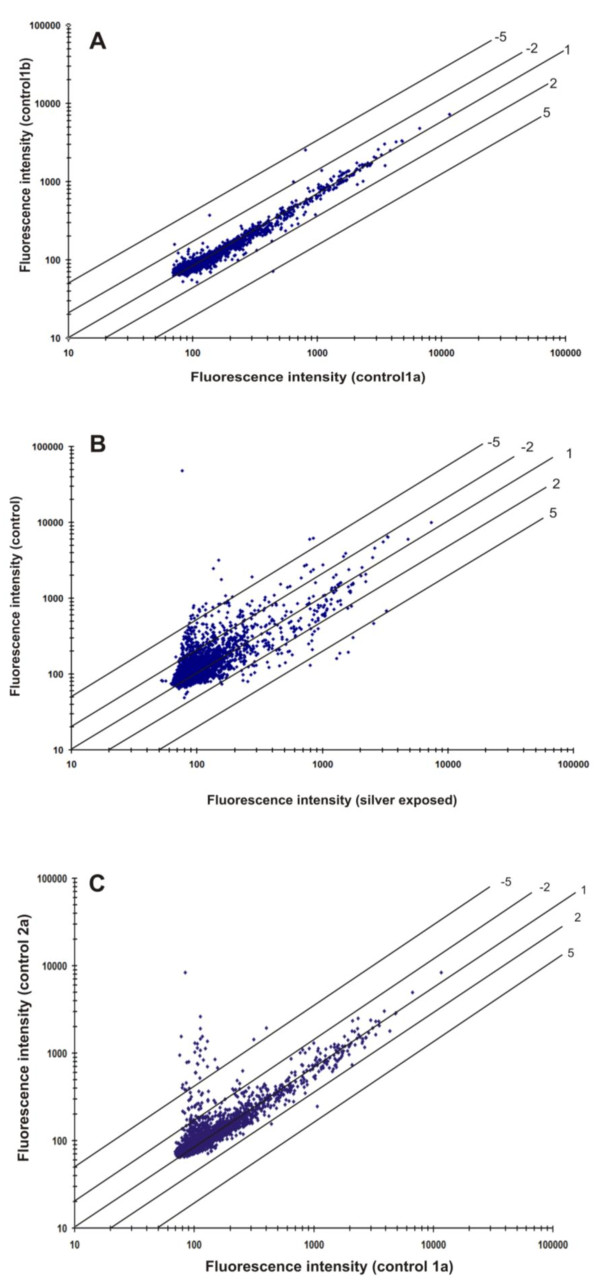
**Scatter plots of normalized spot fluorescence intensities (arbitrary units) from DNA microarrays**. (A) Spot intensities of array hybridizations with two different control samples in duplicates from the *B. cereus *ATCC 14579 (control 1a versus control 1b). (B) Comparison of spot intensities of array hybridizations from control samples (control 1a and AgNO_3 _treated sample test 1a). (C) Spot intensities of the array hybridized from control samples at 30 min (control 1a) and 60 min (control 2a).

Transcriptome analysis was carried out with microarray to study the effect of silver nitrate stress response on the global gene expression in *B. cereus*. These experiments were conducted with a custom-designed 8 × 15 K DNA microarray consisting of oligo probes for coding DNA sequences (CDS's) and intergenic regions (IGR's). The signal intensities obtained from the labelled cRNA of the control cells (30 min) were presented in scatter plot to study the efficacy of the microarray experiments (Figure 3A). Virtually, majority of the spots lie on or close to the 45° line suggesting no difference in gene expression between the biological duplicates. However, some of the genes showed low-intensity signals suggesting a high standard deviation because of background signal (i.e., intensities less than 100 arbitrary units or twice the detection limit are considered as not significantly expressed).

The scatter plot of signal intensities obtained from the cells grown with and without 1 mM AgNO_3 _revealed a clear difference in gene expression profile (Figure 3B). The genes that are up-regulated during silver nitrate stress condition showed signal intensity with at least one fold increase (shown by the upper and lower diagonal lines in Figure 3B). There was also more than a fivefold difference (lower or higher) in signal intensity as indicated by the diagonal lines. The hybridization signal intensity obtained from the control cells at 30 and 60 min (1a and 2a) showed majority of ORFs lying close to the diagonal and few others at the low intensity (Figure 3C). These hybridizations results suggested that over all precision analysis of the microarray using various statistical parameters is of greater accuracy [28].

### Response to silver nitrate stress at transcript level

**Figure 4:**
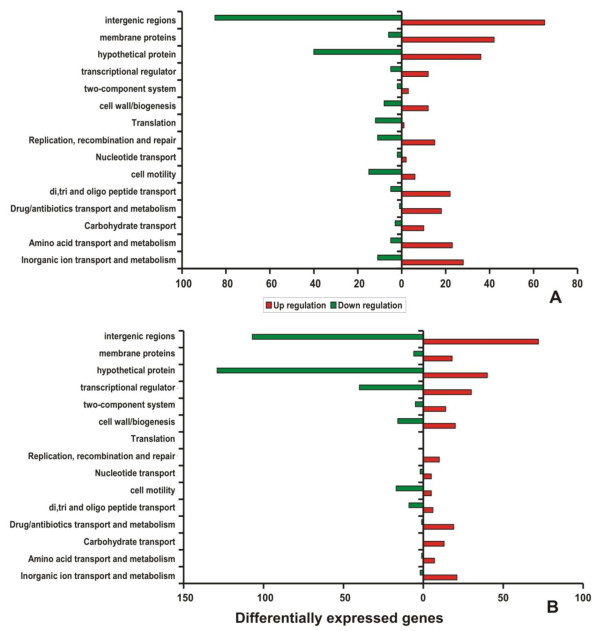
**Histogram of differential expression pattern of genes belonging to COG class**. Number of regulated genes that are differentially expressed after 30 and 60 min exposures to silver nitrate. The red bars represent the number of up-regulated genes and the green bars represent the number of down-regulated genes. A - 30 min and B - 60 min.

Genes showing differential expression in the cells exposed with silver nitrate at 30 and 60 min was compared with the controls. Expression of genes involved in basic cellular processes was classified based on the Clusters of Orthologous Groups (COG). Most of the genes that showed down-regulation during silver nitrate stress conditions were identified to fall under the COG functional classes of cell motility (N), translation (J) and hypothetical proteins. Interestingly, transcripts encoding proteins of transport and metabolism (P) (such as inorganic ion, amino acids, carbohydrate, synthesis of drug/antibiotics and oligopeptide), transcription (K), DNA replication/recombination/repair (L), transcriptional regulators and cell envelope biogenesis/membrane (M) were found to be up-regulated in cells exposed to AgNO_3 _stress (Figure 4A-B). Generally, genes encoding transporters and membrane proteins (e.g. efflux proteins, drug resistance transporters, transcriptional regulators) were found to be up-regulated upon metal ionic stress conditions. These results also confirmed that the induction of osmoprotectant transporters during exposure to silver stress condition. In the genome of *B. cereus*, genes encoding various osmoprotectant transporters were identified. The osmoprotectant gene encoding a proline/betaine transporter belonging to the major facilitator transporter family and a gene encoding a proton-dependent di-, tri- and oligopeptide transporter were among the highly induced genes upon exposure to silver stress. In addition, the genes encoding ABC transporters OpuA [BC2791] and OpuB/OpuC [BC2232] were found to be induced during silver nitrate treatment. Furthermore, the up-regulation of zinc-transporting ATPase [BC0596], cationic Na^+^/H^+ ^antiporters [BC0373 and BC0838] and copper importing ATPase [BC3730] a part of *cop *system were induced upon silver ionic stress. Another Na^+^/H^+ ^antiporter encoded by [BC1612] have been reported to be induced under salt stress but it was observed to be stably expressed upon silver stress. Higher level of expression P-type ATPases in *B. cereus *ATCC14579, a versatile group of ion pumps, has suggested that it contributes to the metal homeostasis in response to silver stress [18]. The *cop *system is a general metal response system that is readily inducible at lower to higher levels of metal stress caused by Cu(II) and Ag(I). Previous report on transcriptional activator like CueR, an Mer R-like was found to respond to Cu(II) and it was also activated by Ag(I) [19,29].

Heat shock proteins (GroEL, GroES, DnaJ and DnaK) are generally induced in microorganisms under various stress conditions [30-32]. But in our study, GroEL [BC0295] alone was up-regulated at the early stages of silver ionic stress. Generally, oxidative stress response genes are involved in response to metal ionic stresses in bacteria. Both, the vegetative catalase-*KatA *[BC1155] and σ^B^-dependent catalase-*Kat*E [BC0863] genes were commonly known to respond to oxidative stresses, but in our study, *KatE *[BC0863] alone was induced upon silver ionic stress and presumed to have essential role in the survival of the cell. In addition, NAD and NADH dependent enzymes especially nitrate reductase [BC2118] and nitroreductase [BC3024] were found to be up-regulated during silver nitrate treatment. The involvement of nitrate reductase in the production of silver nanoparticles has been previously demonstrated [12,33].

The important transcriptional activators of stress sigma factor (σ^B^), *rsbY *[BC1006] and *rsbV *[BC1004] were found to be up-regulated during 30 and 60 min exposure to silver nitrate. The up-regulation of anti-sigma factor antagonist (*rsbY*) indicates the activation of σ^B ^induced global stress response. Most of these induced genes are under the control of alternative sigma factor (σ^B^) in response to variety of stress conditions acting via partner switching mechanisms in Gram-positive bacteria [23,30,34]. Interestingly, silver ion stress is known to induce metabolic pathways associated with amino acid metabolism especially arginine metabolism [21]. The present study has confirmed the over expression of arginine utilization protein [BC0473]. The S-adenosyl methionine -dependent methyl transferase [BC2891] and S-adenosyl homocysteine nucleosidase [BC2503], which are essential for the cellular detoxification of metals were also up-regulated during 30 and 60 min exposure to silver nitrate [35,36]. Summary of gene expression pattern of *B. cereus *ATCC 14579 in response to silver nitrate stress is listed in Additional file 1.

**Figure 5:**
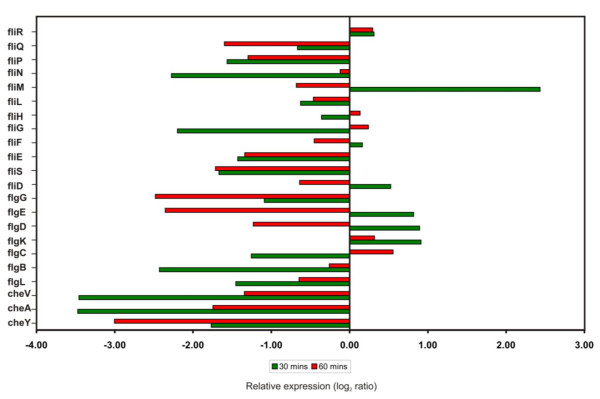
**Histogram of differential expression pattern of motility genes**. Motility-associated genes that were differentially regulated in *B. cereus *ATCC 14579 after exposed to silver ion. The bars indicate the regulated gene after 30 min (green) and 60 min (red) exposure to silver nitrate.

**Figure 6:**
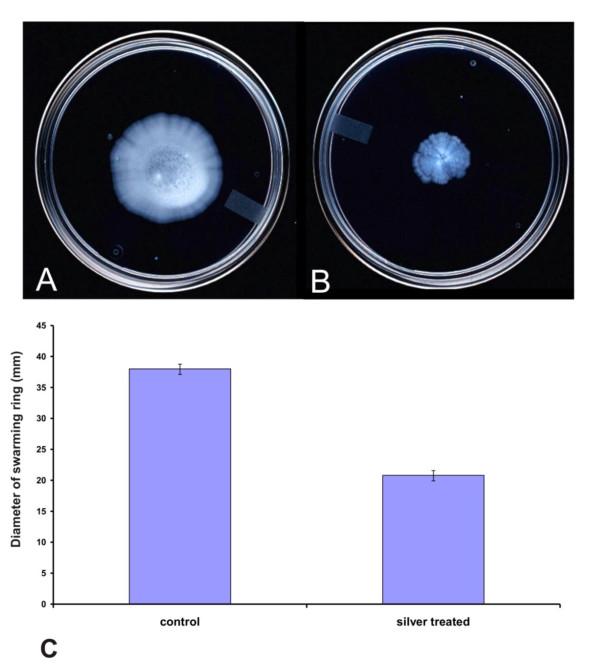
**Influence of silver nitrate stress on motility of *B. cereus***. The *B. cereus *ATCC 14579 was inoculated onto swarming plates without (A) or with exposure to 1 mM silver nitrate (B). After growth at 37°C overnight, plates were observed for swarming capability. (C) The level of motility at 12 h was evaluated as the diameter of the swarming ring in mm. The results presented are the mean value of three independent experiments.

Several genes associated with cell motility are located in a ~45 kb region ranging from locus [BC1625] to locus [BC1671] in the *B. cereus *ATCC14579 genome [23,24]. Most of the flagellar and chemotactic genes were down regulated at transcript level upon silver stress are shown in Figure 5. Most of the chemotaxis related genes (CheY, CheA and CheV), flagellar biosynthetic genes (*fli*P and *fli*Q), hook-associated genes (*flg*L, *flg*E and *fli*E), basal body rod genes (*flg*B and *flgG*) and motor switch genes (*fli*G and *fli*N)] were down-regulated after silver nitrate treatment. Interestingly, there was no change in the expression level of flagellar motor switch (*fli*R), a component of type III secretion system, suggesting that it may not have any role in the ionic stress response. In contrast, most of the flagellar genes exhibited dramatic changes in their expression within 30 min after silver nitrate treatment. But, few of them were down regulated for continued exposure to silver nitrate for 60 min (Figure 5). The genes encoding flagellar components such as basal ring, hook, hook filament junction and cap (*fli*M, *flg*K, *fli*D, *flg*E, *flg*D and *flg*C) were induced in *B. cereus *during early stages of silver stress (30 min). The prolonged silver stress leads to the decreased level of expression of genes involved in biosynthesis of flagellar components. In addition, *fliM *gene is also involved in the generation of energy from transmembrane electrochemical ion gradients, in the form of Na^+^/H^+ ^antiporter (sodium proton motive force) into mechanical energy [37]. At an early stage of silver stress, the genes involved in ion gradients were induced while the prolonged stress repressed the motive force. Our microarray expression profiling study has shown strong evidence for suppression of motility and chemotaxis under silver nitrate stress. This result was supported by the differential expression of chemotaxis and motility related genes in *B. cereus*, *B. subtilis and H. arsenicoxydans *strains in response to various stresses [18,23,38]. Further, the chemotactic behaviour of silver stressed *B. cereus *cells were examined on swarming plates (Figure 6). These results suggested that prolonged exposure of silver nitrate delayed the cell motility.

### Identification of sRNAs in Intergenic regions

**Figure 7:**
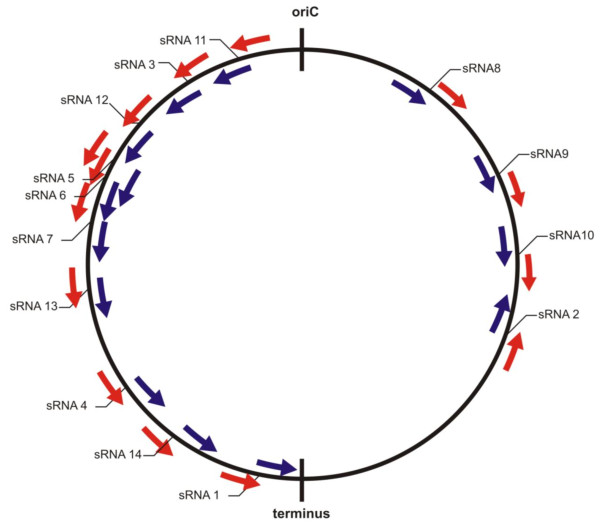
**Distribution of the putative sRNA-encoding genes along the *B. cereus *genome**. The origin and terminus of replication are indicated. sRNA genes on the leading and lagging strands are coloured blue and red, respectively.

**Figure 8:**
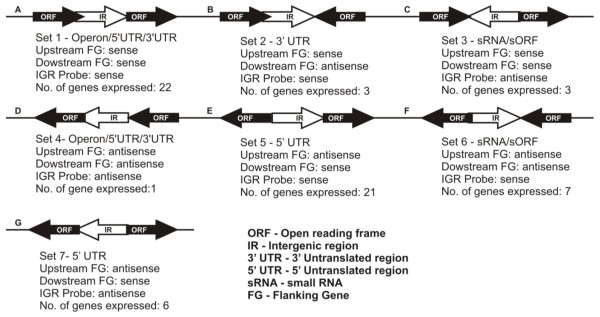
**Schematic representation of the Intergenic region transcript orientations**. Possibility of intergenic transcripts as parallel transcriptional outputs from adjacent mRNAs in the same strand.

Bacterial small and un-translated RNAs serve as crucial regulators of cellular physiology to environmental signals. These sRNAs act against the effectors either at transcription or translation levels. We used genome based microarrays to identify, the expression of candidate sRNAs predicted in the intergenic regions upon silver ionic stress. An intergenic region is assumed to encode a sRNA and that can bind to its specific cognate probes which are complementary to each other. The distribution of sRNA and the possible transcripts from each and every 'empty' intergenic region was modelled from the genome of *B. cereus *shown in Figure 7 and 8. Possible intergenic transcripts as parallel transcriptional outputs from adjacent mRNAs in the same strand are shown in Figure 8A, B &8D. The transcripts expressing from the UTRs of the adjacent genes are shown in Figure 8E &8G. The non-overlapping intact sRNA located within the intergenic region which has diverged orientations with its upstream flanking gene is considered as 'True' sRNA (Figure 8C &8F). The expression pattern of a given transcript was determined in accordance with the computer-assisted algorithm of the Agilent system. In this way, fourteen putative sRNAs collectively from 30 min & 60 min conditions were detected according to their diverging orientations with their adjacent flanking genes. Interestingly, the expression of ten putative sRNAs (sRNA1 to sRNA10) were found under early period (30 min) of exposure to AgNO_3 _stress while other four putative sRNAs (sRNA11 to sRNA14) were found after prolonged (60 min) exposure to AgNO_3 _stress (Table 1). We have compared the locations of the fourteen putative sRNAs with the computational sRNA predictions by sRNA scanner [39]. This observation has confirmed our initial prediction and detection of fourteen putative silver stress responsive sRNAs proposed from this study. These sRNA candidates are presumed to have regulatory roles under silver ionic stress conditions but needs further validations to understand their actual functions. Interestingly, the overexpression of the small RNA binding chaperone protein Hfq [BC1623] was found upon silver ionic stress. It clearly indicates the unknown regulatory cascade of the abundant small RNAs and Hfq protein which needs further validation.

**Table 1:** Summary of RNA transcripts within intergenic regions of *B. cereus *ATCC14579 regulated in response to silver nitrate identified by genome based microarray

sRNA id	Start	End	Length (nucleotides)	Flanking gene id	Strand*	Expression pattern	Relative copy numbers	Primers	
								Start	End
**sRNA1**	2840862	2840921	~60	BC2880/BC2881	<><	↓↑↓	54	2840862	2840921
**sRNA2**	1502573	1502638	~66	BC1553/BC1554	<><	↓↑↓	38	1502576	1502635
**sRNA3**	5024909	5024970	~62	BC5122/BC5123	<><	↓↑↓	36	5024910	5024969
**sRNA4**	3372000	3372189	~190	BC3408/BC3409	<><	↓↑↓	181	3372130	3372189
**sRNA5**	4261246	4261455	~210	BC4317/BC4318	<><	↓↑↓	41	4261395	4261454
**sRNA6**	4219221	4219336	~116	BC4271/BC4272	<><	↓↑↓	56	4219276	4219335
**sRNA7**	4040528	4040740	~213	BC4070/BC4071	<><	↓↑↓	169	4040679	4040738
**sRNA8**	525752	526136	~385	BC0547/BC0548	><>	↓↑↓	142	526073	526132
**sRNA9**	**1106401**	**1106863**	**~463**	**BC1124/BC1125**	**><>**	**↓↑↓**	**44**	**1106797**	**1106856**
**sRNA10**	1381925	1382221	~297	BC1419/BC1420	><>	↓↑↓	183	1382156	1382215
**sRNA11**	5372154	5372598	~445	BC5447/BC5448	<><	↓↑↓	42	5372540	5372599
**sRNA12**	4790423	4791589	~1167	BC4864/BC4865	<><	↓↑↓	19	4791428	4791487
**sRNA13**	3802805	3802907	~103	BC3824/BC3825	<><	↓↑↓	16	38028445	3802904
**sRNA14**	3121534	3121698	~165	BC3151/BC3152	<><	↓↑↓	12	3121639	3121698

*The middle symbol represents the sRNA gene, while the flanking symbol indicates the orientation of the adjacent genes, respectively. Genes present on the strand given in the *B. cereus *genome database are indicated by (>), and genes present on the complementary strand are indicated by (<).

## Conclusions

Chemical and biological methods are used for the synthesis of silver nanoparticles with significantly novel structures, improved physicochemical and biological properties. Bacterial species of *Bacillus*, *Pseudomonas *and certain filamentous fungi are widely used for the biological synthesis of silver nanoparticles. Here, we have studied the transcriptomic and phenotypic responses of the *B. cereus *ATCC 14579 during the biosynthesis of silver nanoparticles. The bacteria can activate various cellular and metabolic adaptive mechanisms to reduce the toxicity and precipitate silver as nano-sized particles. Several microbes are reported to produce silver nanoparticles from the aqueous silver nitrate (~1 mM) and several proteins are expected to play vital role in the detoxification and precipitation of silver nanoparticles. The transcriptome analysis of *B. cereus *ATCC14579 exposed to silver ionic stress was done using whole-genome DNA microarrays. Approximately 10% of the genes were up-regulated but 20% of the genes were down regulated upon silver ionic stress. The SEM along with EDX analysis has revealed the accumulation of Ag nanoparticles in the cell-wall. In general, silver stress which has induced the expression of genes involved in the osmoprotection, transport elements, oxidative stress response and detoxification may have contributed to cross protection. Interestingly, silver ionic stress was observed to delay the cell motility. These characteristic phenotypic assessments can contribute to a better understanding of cellular stress adaptation strategies. Finally, fourteen 'putative' transcripts were found to be induced from the 'empty' intergenic regions upon silver nitrate stress and they are proposed as stress responsive putative sRNAs which could be studied in detail for their role in differential gene expressions.

## Materials and methods

### Bacterial strain culture conditions and SEM analysis

Gram-positive *Bacillus cereus *ATCC 14579 was used as a model system in this study. The strain was routinely grown in LB broth containing yeast extract (10 g/L), peptone (10 g/L) and NaCl (5 g/L) and pH - 8.0) and incubated at 37°C with agitation (200 rpm). The overnight cultures were diluted to 1:100 in 100 ml pre-warmed LB broth and incubated at 37°C, with shaking at 200 rpm until the cells were growing exponentially. When an optical density at 600 nm (OD_600_) of 0.5 - 0.6 was reached, decimal dilutions were prepared using 9 ml of a peptone saline solution and plated on LB agar to determine the viable counts.

To study the effect of silver nitrate exposure, the exponentially grown cells were treated with 1 mM silver nitrate and incubated at 37°C. At intervals, aliquots of control and silver treated cultures were diluted and plated on LB agar plates. The plates were incubated for 24 h at 37°C and the viable cells were expressed as log_10 _colony forming units (CFU). The AgNO_3 _treated cultures and corresponding controls at two different time points (30 and 60 min) were used for gene expression profiling. For each condition of microarray analysis, biological duplicates were prepared.

The accumulation of silver nanoparticles within the *B. cereus *cells after AgNO_3 _treatment for 60 min was recorded with a JEOL Model JSM - 6390LV and JEOL Model JED - 2300 operating at 1 pA to 1 mA with a 3, 8 and 15 nm resolution (JEOL Model JSM - 6390LV and JEOL Model JED - 2300, Tokyo, Japan). Samples were collected by centrifugation (8000 rpm) for 10 min, dried in oven at 60°C overnight. Approximately, 1 g of finely powdered sample was used for SEM-EDX analysis.

### Microarray design

A sense and antisense oligonucleotide microarray slides complementary to the *B. cereus *ATCC14579 genome was custom-designed (obtained from Agilent technologies, USA) using the published DNA sequence [NC_004722] [24]. Antisense oligonucleotide sequences targeting the 'empty' intergenic regions were also designed on the customized genome array. Each oligonucleotide probe was 60 nucleotides in length and was specifically designed using eArray software from Agilent technologies http://earray.chem.agilent.com/earray. Totally, 15,000 probe sets were designed for Gene Expression profiling of *B. cereus *using 8 × 15 k Array AMADID: 23971. It targets the 5234 annotated CDS's, 900 'empty' intergenic regions located on the genome and CDS's encoded by the 21 plasmids.

### Total RNA isolation and cDNA preparation

Bacterial RNA was isolated using the Qiagen RNeasy kit and on-column DNA digestion was carried out according to manufacturer's instructions (Qiagen, Hilden, Germany), additional DNA removal was done by with DNase I (Ambion, Austin, USA). To perform this, the RNA was precipitated and re-constituted in 85 μl of nuclease-free water and then added with 10 μl of 10× DNase I buffer and 5 μl of (1 U/μl) DNase I. The reaction mixture was incubated at 37°C for 30 min and then chilled on ice. A second RNeasy column purification was performed. The RNA isolation protocol for Gram-positive bacteria was followed, in which 3 mg/ml of lysozyme was used to degrade the bacterial cell wall. The RNA quality, purity and integrity were determined using both NanoDropTM 1000 spectrophotometer (Thermo Scientific, Wilmington DE, USA) and RNA 6000 Nano Lab Chips with an Agilent 2100 Bioanalyzer (Agilent Technologies, Santa, CA, USA). Standard methods were used for cDNA synthesis, fragmentation and cyanine3 labelling as per the manufacturer's protocol (Genotypic technologies, Bangalore).

### cRNA preparation, microarray hybridization

The synthesized cDNA was transcribed into cRNA using *in vitro *transcription kit (Agilent Technologies, CA, USA) and labelled with cyanine 3 labelled nucleotide according to manufacturer's protocol and purified with RNeasy Mini columns (Qiagen, Hilden, Germany). The quality of the labelled cRNA sample was verified by the total yield and specificity calculated based on NanoDrop ND-1000 spectrophotometer (Thermo Scientific, Wilmington DE, USA). Labelled cRNAs with specificity greater than 7 were considered as of high quality and taken for hybridization using the *in situ *hybridization kit plus (Agilent Technologies, Santa, CA, USA). Then, the arrays were incubated at 65°C for 16 h in Agilent's microarray hybridization chambers and the hybridized slides were washed according to the manufacturer's protocol.

### Image processing and Data analysis

Arrays were scanned at 5 μm resolution using the Agilent Microarray Scanner G Model G2565BA and images were saved as TIFF format. Data extractions from images were performed with the Feature Extraction software v 10.5.1 and GeneSpring GX version 11.0 software (Agilent Technologies, Santa, CA, USA). Normalization of the data was carried out by GeneSpring GX using the percentile shift normalization which is a global normalization, where the locations of all the spot intensities in an array are adjusted. This normalization takes each column in an experiment independently, and computes the *n*^th ^percentile of the expression values for this array, across all spots (where n has a range from 0-100 and n **_= _**75 is the median). It subtracts this value from the expression value of each entity and normalized to specific samples. After normalization, controls were removed and replicates corresponding to the same genes within each slide were averaged, provided that there were at least two replicates left after the initial filtering procedure. Otherwise, the gene entry was removed. The average values were compared across slides. The model was evaluated on the basis of that *P *values computed using a false discovery rate correction. Genes were considered regulated at a statistically significant level if they had a *P *value below 0.05 and a ratio of ± 1 cut-off relative to the reference cDNA. Several controls were employed to minimise the technical and biological variations and ensure the quality of the data: 1) each ORFs were present in duplicates in each array 2) array slides were prepared in duplicates for each experiments and 3) two independent RNA batches from each condition were used.

### Microarray data submission and accession numbers

The microarray data derived from this study have been deposited in the National Center for Biotechnology Information (NCBI) Gene Expression Omnibus http://www.ncbi.nlm.nih.gov/geo and are accessible through GEO accession number - [GSE26043].

### Motility Assay

Cells from exponentially grown cultures with and without 1 mM AgNO_3 _treatment for 60 min were recovered by centrifugation at 10,000 × g at 4°C for 1 min. The sample volume of the silver stressed cells was adjusted to equivalent cell density and inoculated into LB-soft agar (0.4% agar) plates and incubated at 37°C overnight.

## Competing interests

The authors declare that they have no competing interests.

## Authors' contributions

MMGB has made substantial contributions to conception, design, acquisition, collection, analysis and interpretation of data and wrote the paper. JS carried out the computational analyses of sRNA. PG provided research support and involved in drafting and revising the manuscript critically for important intellectual content. All authors read and approved the final manuscript.

## Supplementary Material

Additional file 1 **Supplemental information to manuscript**. Expression patters of genes responding to silver nitrate stress. Click here for file
