# Supplementary material for: Global transcriptome analysis of Bacillus cereus ATCC 14579 in response to silver nitrate stress
Source: J Nanobiotechnology. 2011 Nov 10;9:49. doi: 10.1186/1477-3155-9-49 (PMC3247866; doi:10.1186/1477-3155-9-49)
Supplement: Additional file 1 — Supplemental information to manuscript. Expression patters of genes responding to silver nitrate stress. [file 1477-3155-9-49-S1.DOC]

| **Gene** | **Annotation** | **Intensity Ratio** | |
| --- | --- | --- | --- |
|  |  | **30 min** | **60 min** |
| BC4545 | Ferrichrome transport system permease protein fhuB | 2.27 | 6.93 |
| BC2532 | S-adenosylmethionine-dependent methyltransfereases | 1.17 | 5.99 |
| BC0362 | hypothetical protein | 2.93 | 5.86 |
| BC5206 | hypothetical protein | 2.45 | 5.64 |
| BC5072 | ABC transporter ATP-binding protein | 3.36 | 5.12 |
| BC3679 | multidrug ABC transporter permease,ATP-binding protein; | 1.94 | 4.73 |
| BC2912 | alkanesulfonates transporter ATP-binding protein; | 1.93 | 4.48 |
| BC3024 | NAD(P)H nitroreductase | 2.10 | 4.12 |
| BC1006 | Sigma factor sigB regulation protein rsbY | 2.45 | 3.53 |
| BC0871 | Multidrug ABC transporter permease | 1.8 | 3.49 |
| BC0908 | oligopeptide transport system permease protein oppB | 3.46 | 3.47 |
| BC1337 | MarR family transcriptional regulator | 3.09 | 3.24 |
| BC5418 | major facilitator transporter | 2.17 | 3.24 |
| BC1186 | hypothetical protein | 2.6 | 3.12 |
| BC2978 | M20 Peptidase | 1.44 | 2.73 |
| BC4710 | hypothetical protein | 2.21 | 2.67 |
| BC2961 | Sugar transport system permease protein; | 1.65 | 2.41 |
| BC3426 | Putative RNA polymerase sigma factor sigL | 2.02 | 2.2 |
| BC0356 | Sigma 54 dependent transcriptional activator | 1.47 | 2.13 |
| BC4691 | Thioredoxin | 0.67 | 2.13 |
| BC4361 | Ferrichrome ABC transporter ATP-binding protein; | 1.12 | 2 |
| BC2118 | Respiratory nitrate reductase alphachain | 0.18 | 1.02 |
| BC2119 | Respiratory nitrate reductase betachain | 0.21 | 1.22 |
| BC0298 | Two-component response regulator | 18.6 | 2.78 |
| BC2444 | transcription state transcriptional regulator AbrB | 15.63 | 2.42 |
| BC3310 | major facilitator transporter | 10.2 | 2.33 |
| BC2069 | Acetyltransferase | 9.63 | 2.3 |
| BC0403 | Glutamine transport ATP-binding protein glnQ | 7.23 | 2.29 |
| BC0712 | Phosphate ABC transporter permease | 6.5 | 2.06 |
| BC0865 | arginine,ornithine antiporter | 6.29 | 2.04 |
| BC0936 | two-component sensor kinase citS | 5.47 | 1.82 |
| BC4544 | Ferrichrome ABC transporter ATP-binding protein | 5.21 | 1.77 |

**Additional File 1 Expression patterns of genes responding to silver nitrate treatment at 30 and 60 min**

| BC1964 | homoserine dehydrogenase | 5.14 | 1.74 |
| --- | --- | --- | --- |
| BC3634 | Acetyltransferase | 5.13 | 1.71 |
| BC4849 | two-component response regulator vanR | 5.12 | 1.69 |
| BC4940 | ABC transporter ATP-binding protein | 4.97 | 1.67 |
| BC5295 | NADH dehydrogenase subunit J | 3.86 | 1.56 |
| BC1438 | two-component sensor protein yvqE | 3.8 | 1.55 |
| BC1360 | bacitracin transport permease protein BCRB | 3.72 | 1.53 |
| BC3693 | PadR family transcriptional regulator | 3.45 | 1.37 |
| BC3168 | xanthine dehydrogenase molybdopterin-binding subunit | 3.36 | 1.36 |
| BC3128 | MarR family transcriptional regulator | 3.33 | 1.35 |
| BC0295 | Chaperonin GroEL | 3.33 | 1.34 |
| BC0980 | TetR family transcriptional regulator | 3.16 | 1.31 |
| BC2457 | 4'-phosphopantetheinyl transferase | 3.11 | 1.26 |
| BC2494 | aminoglycoside N6'-acetyltransferase | 3.07 | 1.26 |
| BC2549 | GntR family transcriptional regulator | 3.00 | 1.22 |
| BC2288 | Acyl-CoA dehydrogenase | 2.84 | 1.22 |
| BC4075 | D-alanyl-D-alanine carboxypeptidase | 2.83 | 1.2 |
| BC1719 | MecI family transcriptional regulator | 2.83 | 1.2 |
| BC0410 | Crp family transcriptional regulator | 2.77 | 1.18 |
| BC0473 | arginine utilization regulatory protein rocR | 2.76 | 1.17 |
| BC1155 | Catalase | 2.72 | 1.16 |
| BC1589 | putative N-acetylgalactosaminyl-diphosphoundecaprenol glucuronosyltransferase | 2.65 | 1.12 |
| BC2631 | ArsR family transcriptional regulator | 2.55 | 1.11 |
| BC0586 | two-component response regulator | 2.54 | 1.08 |
| BC5353 | two-component sensor kinase YocF | 2.53 | 1.07 |
| BC0209 | oligopeptide transport ATP-binding protein oppD | 2.53 | 1.04 |
| BC4845 | L-seryl-tRNA(Sec) selenium transferase | 2.43 | 1.03 |
| BC2961 | sugar transport system permease protein | 2.35 | 1.00 |
| BC1396 | branched-chain amino acid aminotransferase | 2.34 | 0.96 |
| BC2631 | ArsR family transcriptional regulator | 2.3 | 0.96 |
| BC0290 | ABC transporter ATP-binding protein uup | 2.25 | 0.94 |
| BC2723 | D-amino acid dehydrogenase small subunit | 2.05 | 0.84 |
| BC0567 | SN-glycerol-3-phosphate transport ATP-binding protein ugpC | 2.02 | 0.83 |

| BC3409 | MarR family transcriptional regulator | 2.01 | 0.81 |
| --- | --- | --- | --- |
| BC4222 | GntR family transcriptional regulator | 1.99 | 0.8 |
| BC0871 | multidrug ABC transporter permease, ATP-binding protein | 1.92 | 0.76 |
| BC0208 | oligopeptide transport system permease protein oppC | 1.86 | 0.76 |
| BC1359 | bacitracin transport ATP-binding protein bcrA | 1.8 | 0.68 |
